# Nutritional Intervention Strategies Using Dietary Antioxidants and Organic Trace Minerals to Reduce the Incidence of Wooden Breast and Other Carcass Quality Defects in Broiler Birds

**DOI:** 10.3389/fphys.2021.663409

**Published:** 2021-04-06

**Authors:** Vivek A. Kuttappan, Megharaja Manangi, Matthew Bekker, Juxing Chen, Mercedes Vazquez-Anon

**Affiliations:** Novus International, Inc., St. Charles, MO, United States

**Keywords:** myopathy, wooden breast, skin scratches, tibial head lesions, nutrition, minerals, antioxidants

## Abstract

Wooden breast (WB) is a degenerative myopathy seen in modern broiler birds resulting in quality downgrade of breast fillets. Affected filets show increased toughness both before as well as after cooking and have decreased water holding capacity and marinade pick up compared to normal fillets. Although the exact etiology is unknown, the circulatory insufficiency and increased oxidative stress in the breast muscles of modern broiler birds could be resulting in damage and degeneration of muscle fibers leading to myopathies. Three independent experiments were conducted to evaluate the effect of various dietary interventions on the incidence of WB when birds are exposed to oxidative stress associated with feeding oxidized fat and mild heat stress. Feed additives such as dietary antioxidant [Ethoxyquin (ETX)], mineral methionine hydroxy analog chelate (MMHAC) of Zn, Cu, and Mn, and organic selenium (Org Se) were tested at recommended levels. In experiment 1, ETX reduced (*P* < 0.05) the incidence of severe WB induced by oxidized fat diet. The magnitude of improvement in percentage of normal (no WB) filets and reduction in muscle lipid peroxidation was greater (*P* < 0.05) when ETX and MMHAC were fed together as shown by experiment 2. In birds exposed to mild heat stress (Experiment 3), feeding MMHAC by itself reduced (*P* < 0.05) tissue damage by reducing incidence of tibial head lesions, skin scratches, breast blisters, in addition to increasing the incidence of normal (no WB) fillets. When MMHAC was combined with ETX and Org Se, further improvement (*P* < 0.05) in normal (no WB) filets was observed. In summary, under different oxidative stress conditions, dietary intervention programs that contain ETX, MMHA-Zn, -Cu, and -Mn and Org Se can improve performance and increase carcass integrity, reducing problems, such as WB, either independently or with additive effect. This effect is most likely attained by simultaneously improving the exogenous and endogenous antioxidant status, reducing oxidative stress, and improving tissue healing process of the bird.

## Introduction

Wooden breast (WB) is a breast myopathy affecting modern broiler birds resulting in downgrading of breast filets causing economic loss to the poultry industry. WB is defined as the occurrence of toughness in raw breast filets and the severity varies from toughness only at the cranial region to a generalized toughness affecting the whole filet (Sihvo et al., 2014; Tijare et al., 2016). On average, the incidence of the condition reported in different publications is around 20% although, the reports may be confounded by the classification criteria and the absolute incidence across industry could vary tremendously (Petracci et al., 2019). Histologically, WB is characterized by multifocal and multiphasic degeneration and regeneration of muscle fibers, variability in shape, size, and diameter of the muscle fibers, increased deposition of fibrous tissue and lipid, infiltration of macrophages and lymphocytes, edema, vasculitis, and perivascular infiltrations in veins (Sihvo et al., 2014, 2017; Papah et al., 2017; Soglia et al., 2017; Petracci et al., 2019). These tissue changes are reported to have resulted in reduced protein as well as lipid content (Soglia et al., 2016a,b; Wold et al., 2017; Cai et al., 2018), and increased moisture (Soglia et al., 2016a,b; Wold et al., 2017; Cai et al., 2018) as well as collagen (Soglia et al., 2016a,b) contents in severe WB filets (Petracci et al., 2019). Various studies showed that severe WB results in increased toughness, drip loss, reduced marinade absorption and retention, also increased moisture loss in cooking of meat products (Mudalal et al., 2015; Chatterjee et al., 2016; Soglia et al., 2016b; Tijare et al., 2016; Dalle Zotte et al., 2017; Kuttappan et al., 2017a; Aguirre et al., 2018; Bowker et al., 2018; Brambila et al., 2018). Interestingly, the meat batters and meat balls produced from WB meat had inferior protein gelation and product texture compared to products from normal filets (Xing et al., 2017a; Chen et al., 2018). Based on its impact on the quality of fillets, the estimated economic loss due to WB condition could be $200 million to $1 billion per year for the United States poultry industry (Kuttappan et al., 2016; Huang and Ahn, 2018).

Despite the large number of studies conducted in WB over the past decade, the exact etiology of the condition is not clear yet. Various studies reported that the condition is associated with birds having faster growth rate or heavier filets (Mudalal et al., 2015; Kuttappan et al., 2017a), although the incidence of this condition can occur on birds of any size or growth rate. Furthermore, Soglia et al. (2016a) reported that severe WB is characterized by an increased oxidative stress in muscle tissue measured using thiobarbituric acid reactive substances (TBARS) and protein carbonyl levels. Several studies conducted using “omics” approach have revealed that incidence of WB is associated with signs of vascular disturbances, hypoxia, increased oxidative stress and altered metabolic pathways (Mutryn et al., 2015; Abasht et al., 2016; Kuttappan et al., 2017b; Papah et al., 2018). Arguably, one of the more popular hypothesis on the etiology of WB or similar myopathies is that broilers with higher rate of hypertrophic muscle growth results in increased metabolic and circulatory demand leading to increased risk of accumulation of metabolic waste products (such as oxygen free radicals) in muscle tissues resulting in increased oxidative stress (Petracci et al., 2019). The accumulated free radicals are highly reactive and can damage DNA, RNA, proteins, and lipids present in the muscle cells (Surai, 2015) causing inflammation and metabolic disturbances, eventually causing degeneration of muscle fibers. Once the damage caused by increased oxidative stress is beyond the regenerative capacity of the muscle cells, the result is an accumulation of fibrous tissue and fat leading to myopathies such as WB (Petracci et al., 2019).

Various intervention strategies are already being considered to reduce the incidence of WB in the industry. These strategies can be broadly classified into growth-rate-related and antioxidant-based approaches. Growth-rate-related approaches focus on manipulating or slowing down the birds during the entire or a part of the growth period and thus reduce myopathies, utilizing the knowledge of positive correlation between growth rate and incidence of myopathies (Kuttappan et al., 2016). Dietary restriction, reduced nutrient density, and lysine reduction during growth phase have been studied with variable success in different studies (Kuttappan et al., 2013a; Cruz et al., 2016; Bodle et al., 2018; Livingston et al., 2018; Meloche et al., 2018a,b,c,d). However, if not applied properly, these approaches have a risk of compromising the performance in addition to reduction in myopathies. On the other hand, the antioxidant-based approaches such as the use of dietary arginine, vitamin C, selenium, and trace minerals (Sirri et al., 2016; Bodle et al., 2018; Cemin et al., 2018) have been evaluated to reduce myopathies. Similar to the growth-rate-related approach, the benefits from the antioxidant-based approach are also variable and sometimes confounded by its effect on growth rate, slaughter weight and/or breast yield which makes it difficult to draw any meaningful conclusions. It is well known that factors such as oxidized fat in the diet and heat stress, which are very relevant to commercial poultry production in certain world areas, can also lead to increased oxidative stress in tissues (Jensen et al., 1997; Willcox et al., 2004; Slimen et al., 2014; Delles et al., 2015; Surai, 2015; Akbarian et al., 2016; Wang et al., 2018), and eventually could result in increased myopathies. In fact, dietary antioxidants, vitamins, and bioavailable trace minerals such as zinc, copper, manganese, and selenium have been proven to reduce oxidative stress in animal tissue (Dibner et al., 1996; Avanzo et al., 2001; Xiang et al., 2002; Surai, 2015). So, the main objective of the present study was to evaluate the effect of various dietary antioxidant based (synthetic antioxidants) or endogenous antioxidant support (through highly bioavailable trace minerals such as zinc, copper, manganese, and selenium) approaches on the incidence of WB in broilers under oxidative challenge (oxidized fat or heat stress) and identify an appropriate strategy which may reduce WB without compromising performance in broiler birds.

## Materials and Methods

### Experiment 1

The study was conducted at Novus International, Inc., Green Acres Experimental Farm, Montgomery, MO, United States and all the procedures were approved by the Animal Use Committee. Total of 792 1-day-old male broilers were allocated to four diet treatments, with 18 pen replicates per treatment with 11 males per pen, in a randomized complete block design. All the treatment groups received the same corn-soybean meal-based basal diet (Table 1) with Zn, Cu, and Mn in the form of sulfates at 110-20-120 ppm, respectively, with respective treatment feed additives in different phases–Starter (0–11 days), Grower (11–25 days), Finisher-1 (25–39 days), Finisher-2 (39–46 days), and withdrawal (46–58 days). Oxidized vegetable fat/oil used for the diet was prepared by bubbling air through the fat at 92°C for 24 h (method Cd 12-57; AOCS., 1997) to contain peroxide value of 225 mEq/kg so that the final starter diet would have ∼5–5.5 mEq/kg diet, and the grower and finisher diets will have ∼7–7.5 mEq/kg. Antioxidant used for respective treatments in the diets was SANTOQUIN^®^ M6 [with 66.6% ethoxyquin (ETX)] added at 188 ppm in the treatments diets to get a final concentration of 125 ppm ETX in diet. All diets were conditioned and pelleted at 85°C for 30 s and fed as crumbles during starter phase and as pellets for the remainder of grow out. Four treatments in the study were: 1. Fresh fat without antioxidants (FrF), 2. Fresh fat with antioxidant (FrF + ETX) 3. Oxidized fat without antioxidant (OxF) and 4. Oxidized fat with antioxidant (OxF + ETX). Other than the differences in treatments, all diets satisfied the nutritional requirements described by Aviagen breed recommendations. On 59 days, two birds per pen were randomly selected to collect blood and muscle tissue for plasma enzymes and TBARS measurement, respectively. All the remaining birds were processed according to standard commercial style system as described by Kuttappan et al. (2013a). At 10 h before processing, all birds were withheld feed while having *ad libitum* access to water. At the first stage of processing, birds were electrically stunned, the left carotid artery and jugular vein were manually severed, bled out, carcasses were soft scalded, defeathered, and eviscerated. The carcasses were then pre-chilled at 12°C for 15 min followed by further chilling for 90 min at 1°C in immersion chilling tanks. The carcasses were then removed from the chilling tanks and stored at 4°C overnight until deboned. Live weight, carcass weight, filet weight, and abdominal fat were recorded. Filets were scored for different degrees of WB according to the procedure by Tijare et al. (2016) and Kuttappan et al. (2016). Briefly, degree of hardness of whole raw breast filets was evaluated (WB) and categorized as: 0 = filets that were flexible throughout (normal); 1 = filets that were hard mainly in the cranial region but flexible otherwise (mild); 2 = filets that were hard throughout but flexible in mid to caudal region (moderate); 3 = filets that were extremely hard and rigid throughout from cranial region to caudal tip (severe).

**TABLE 1:** Basal diet for different phases for Experiment 1 and 2.

**Ingredient (%)**	**Starter**	**Grower**	**Finisher 1**	**Finisher 2**	**Withdrawal**
Corn	54.22	57.94	66.52	68.4	68.76
Soybean meal	38.78	34.52	26.06	24.3	24.15
Dicalcium phosphate	1.9	1.72	1.53	1.44	1.38
Limestone	0.95	0.89	0.84	0.82	0.78
Salt	0.31	0.24	0.33	0.24	0.25
Sodium bicarbonate	0.3	0.42	0.29	0.47	0.47
^1^MHA^®^84%	0.3	0.26	0.27	0.22	0.18
^2^Mineral premix	0.2	0.2	0.2	0.2	0.2
L-LYSINE HCL 78%	0.14	0.11	0.21	0.18	0.13
Choline Cl-60%	0.05	0.07	0.09	0.09	0.09
^3^Coban 90	0.05	0.05	0.05	0.05	0.05
Threonine	0.1	0.05	0.08	0.06	0.03
^4^Vitamin premix	0.05	0.05	0.05	0.05	0.05
^5^Sand	0.15	0.15	0.15	0.15	0.15
^6^Soybean oil	2.5	3.33	3.33	3.33	3.33
Total	100	100	100	100	100
**Calculated nutrient content**					
ME (Kcal/Kg)	3030	3120	3201	3226	3230
CP (%)	23.62	21.83	18.67	17.91	17.78
dLys (%)	1.28	1.15	1.02	0.95	0.91
dMet (%)	0.59	0.54	0.51	0.46	0.43
dSAA (%)	0.95	0.87	0.80	0.74	0.71
dThr (%)	0.87	0.77	0.68	0.64	0.61
dCys (%)	0.36	0.33	0.29	0.28	0.28
dArg (%)	1.44	1.32	1.09	1.04	1.04
Calcium (%)	0.96	0.88	0.79	0.75	0.72
Phosphorus (%)	0.48	0.44	0.39	0.37	0.36

*Proximate analysis was done for all the diets and confirmed the nutrient value matched the table values.*

*^1^DL-Methionine Hydroxy Analog Calcium, Novus International, Inc., St Charles, MO, United States. For experiment 2, ∼0.06% was reduced in the basal diet for all phase to account the amount coming from mineral premix.*

*^2^Trace mineral premix*

• Experiment 1: Added at this rate yields 120 mg manganese (sulfate), 110 mg zinc (sulfate), 35 mg iron (sulfate), 20 mg copper (sulfate), iodate (calcium iodate) 1.25 mg per kg of diet. The premix contained calcium carbonate as carrier and less than 1% mineral oil.

• Experiment 2: For treatments OxF and OxF + ETX, premix added at this rate had 120 mg manganese (sulfate), 110 mg zinc (sulfate), 35 mg iron (sulfate), 20 mg copper (sulfate), iodate (calcium iodate), and 575.4 mg MHA per kg of diet. For treatments OxF + MMHAC and OxF + MMHAC + ETX, premix added at this rate had 40 mg manganese (MINTREX^®^), 40 mg zinc (MINTREX^®^), 35 mg iron (sulfate), 25 mg copper (MINTREX^®^), and iodate (calcium iodate) per kg of diet.

*^3^Active drug ingredient monensin sodium (Elanco Animal Health, Greenfield, IN, United States). For the prevention of coccidiosis caused by *Eimeria necatrix*, *Eimeria tenella*, *Eimeria acervulina*, *Eimeria brunetti*, *Eimeria mivati*, and *Eimeria maxima*.*

*^4^Vitamin premix: for Experiment 1 and 2, added at this rate yields 11574 IU vitamin A, 4125 ICU vitamin D, 125 IU vitamin E, 3.1 mg vitamin K, 0.019 mg B12, 10.0 mg riboflavin, 68.8 mg niacin, 18.8 mg pantothenic acid, 1.87 mg folic acid, 4.4 mg pyroxidine, 3.13 mg thiamine, 0.188 mg biotin per kg diet. ^5^Sand was used as a filler*

• Experiment 1: ETX-Ethoxyquin [SANTOQUIN^®^ M6 at 188 ppm (Ethoxyquin-125 ppm)]. SANTOQUIN^®^ M6 has 66.6% ethoxyquin and was added at 0.0188% in the FrF + ETX and OxF + ETX diets replacing the same amount of sand.

• Experiment 2: ETX- Ethoxyquin [SANTOQUIN^®^ M6 at 188 ppm (Ethoxyquin-125 ppm)]. SANTOQUIN^®^ M6 has 66.6% ethoxyquin and was added at 0.0188% in the OxF + ETX, OxF + MMHAC + ETX diets replacing the same amount of sand.

*^6^Soybean oil was used as source of fat.*

• Experiment 1: Oxidized soy oil to contain 225 meq peroxide/kg so that the final starter diet to have ∼5–5.5 mEq/kg diet, and the grower and finisher diets to have ∼7–7.5 meq peroxide/kg for OxF and OxF + ETX, while FrF and FrF + ETX received fresh soybean oil.

• Experiment 2: All diets received oxidized soy oil to contain 225 meq peroxide/kg so that the final starter diet to have ∼5–5.5 mEq/kg diet, and the grower and finisher diets to have ∼7–7.5 meq peroxide/kg.

### Experiment 2

The study was conducted at Novus International, Inc., Green Acres Experimental Farm, Montgomery, MO, United States and all the procedures were approved by the Animal Use Committee. The experimental design consisted of four treatments, 18 pen replicates per treatment with 15 males per pen. Total of 1080 day old male broilers were allocated to one of the four diet treatments in a randomized complete block design with Starter (0–12 days), Grower (12–26 days), and Finisher (26–40days) periods. Feed and bird weights were taken on 0, 12, 26, and 41 days. All birds in the study received a corn-soy-based basal diet (Table 1) with oxidized vegetable fat/oil along with the respective treatments. Four treatment groups were: (1) Treatment containing inorganic trace minerals without any feed additives (OxF), (2) Oxidized fat with antioxidant (OxF + ETX), (3) Oxidized fat with inorganic trace minerals replaced by reduced levels of mineral methionine hydroxy analog chelate (MMHAC) trace minerals (OxF + MMHAC), (4) Oxidized fat with both antioxidant and MMHAC trace minerals (OxF + ETX + MMHAC). Antioxidant used in the study was SANTOQUIN^®^ M6 (Novus International, Inc.) at the levels mentioned in experiment 1. Zn, Cu, and Mn in the form of inorganic trace minerals (sulfates) at 110-20-120 ppm and MMHAC (MINTREX^®^, Novus International, Inc.) at 40-25-40 ppm, respectively, for respective treatments were used. All diets, satisfied the nutritional requirements described by Aviagen breed recommendations, were conditioned and pelleted at 85°C for 30 s and fed as crumbles during starter phase and as pellets for the rest of the grow out. Body weight, feed intake, and mortality were recorded until 41 days and cumulative feed conversion corrected for mortality (cFCR) as well as performance index (cPERFIDX) were calculated as described by Anjos et al. (2012). On day 41, two randomly selected male birds were removed from each pen and processed using a standard commercial style system. From the same birds, blood and muscle samples were collected for serum lactate dehydrogenase (LDH) and muscle TBARS measurement as described below. Filets were scored for different degrees of WB according to the procedure by Tijare et al. (2016) and Kuttappan et al. (2016).

### Experiment 3

The study was conducted at Bangkok Animal Research Center Co., Ltd. in Thailand and all the procedures were approved by Internal Ethics Committee. A total of 234 1-day-old male broilers were assigned to 3 dietary treatments with 6 replicates and 13 birds per replicate. All diets satisfied the nutritional requirements described by Aviagen breed recommendations. Corn and soybean meal basal diet formed the basis of the ration (Table 2). The treatments in the study were: (1) CON-ITM with sulfate of zinc (110 ppm), copper (6 ppm), and manganese (120 ppm) with inorganic selenium (0.3 ppm); (2) MMHAC (MINTREX^®^, Novus International, Inc.) with MMHAC sources of zinc (50 ppm), copper (10 ppm), manganese (60 ppm) with inorganic selenium (0.3 ppm); (3) MMHAC + Org Se + ETX with MMHAC sources of zinc (50 ppm), copper (10 ppm), manganese (60 ppm) with organic selenium (Org Se) (ZORIEN^®^ SeY, Novus International, Inc., added at 0.3 ppm) and ETX at 125 ppm of final concentration. Diets were mixed by a double ribbon horizontal mixer. Mash feeds were processed at 82°C conditioning temperature and fed in crumble form for the first 14 days, and in pellet form thereafter until finishing the 35 days test period. The diets were identical and nutritionally equivalent across all parameters outside of the minerals and antioxidant. All birds were grown up to age of 35 days and the heat in the shed was increased to 30°C in the growing period from 21–35 days which would mimic the mild heat stress situation common in South East Asia and many other poultry rearing regions. On 35 days, randomly selected birds (*n* = 24/treatment) were processed using a standard commercial type processing system and live weight, carcass weight, filet weight, and abdominal fat were recorded. Filets were scored for different degrees of WB according to Tijare et al. (2016) and Kuttappan et al. (2016). Tibial strength and severity (Score 0-normal to Score 3-severe) of tibial head necrosis lesions (Wideman et al., 2014) were recorded. Presence or absence of breast and skin scratches were also scored.

**TABLE 2:** Basal diet for different phases for Experiment 3.

**Ingredient (%)**	**Starter**	**Grower**	**Finisher**
Corn	55.38	57.70	61.83
Soybean meal	36.32	33.29	28.63
Soybean oil	3.18	4.37	5.31
Monodicalcium phosphate	2.15	1.91	1.70
Limestone	1.02	0.96	0.85
Salt	0.20	0.23	0.24
^1^Pelex^®^ Dry	0.30	0.30	0.30
^2^BS Premix	0.20	0.20	0.20
^3^MHA^®^84%	0.39	0.33	0.30
L-Lysine HCl	0.30	0.23	0.21
L-Threonine	0.18	0.13	0.10
Sodium bicarbonate	0.25	0.22	0.21
Choline Chloride 60%	0.08	0.07	0.08
^4^Salinomycin 12% (Sacox)	0.05	0.05	0.05
Total	100.00	100.00	100.00
**Calculated nutrient content**			
ME (Kcal/Kg)	3,000	3,100	3,200
CP (%)	23.0	21.5	19.5
dLys (%)	1.28	1.15	1.02
dMet (%)	0.64	0.57	0.53
dCys (%)	0.31	0.30	0.27
dThr (%)	0.86	0.77	0.68
dArg (%)	1.32	1.23	1.10
Calcium (%)	0.96	0.87	0.78
Total phosphorus (%)	0.84	0.77	0.71
Phosphorus (%)	0.48	0.44	0.39

*Proximate analysis was done for all the diets and confirmed the nutrient value matched the table values.*

*^1^Pelex^®^ is an advanced-generation, synthetic resin that is effective at low inclusion levels as a livestock feed/pellet binder.*

*^2^Trace mineral premix: All treatments received 20 mg iron, and 1.2 mg iodine. In addition to that, CON–ITM received 110 mg zinc sulfate, 6 mg copper sulfate, 120 mg manganese sulfate, and 0.3 mg sodium selenite; MMHAC received 50, 10, and 60 mg of MINTREX^®^ Zn, MINTREX^®^ Cu, and MINTREX^®^ Mn, respectively, and 0.3 mg sodium selenite; MMHAC + Org Se + ETX received 50, 10, and 60 mg of MINTREX^®^ Zn, MINTREX^®^ Cu, and MINTREX^®^ Mn, respectively, 0.3 mg ZORIEN^®^ SeY (A special strain of *Saccharomyces cerevisiae* enriched with selenium in an organic highly bioavailable form) and 125 mg of ethoxyquin (SANTOQUIN^®^ M6 at 188 ppm) per kg of diet.*

*^3^DL-Methionine Hydroxy Analog Calcium, Novus International, Inc., St Charles, MO, United States.*

*^4^Salinomycin sodium concentrations ranging from 40 to 60 g/ton (0.0044–0.0066%) for the prevention of coccidiosis in broiler, roaster and replacement chickens caused by *Eimeria tenella*, *Eimeria necatrix*, *Eimeria acervulina*, *Eimeria maxima*, *Eimeria brunetti*, and *Eimeria mivati*.*

### Measurement of Serum/Plasma Markers and Muscle TBARS

Plasma LDH and creatine kinase (CK) activity were measured using LDH activity assay kit and CK activity assay kit from Sigma (St. Louis, MO, United States) as described in the manuals. Serum TBARS was measured using TBARS assay kit (Ann Arbor, MI, United States) from Cayman Chemical as described in the manual.

### Statistical Analysis

Performance, tibial strength, and processing weight as well as yield data were analyzed using a one-way or two-way ANOVA, according to the experimental design, using the SAS GLM procedure and means were separated at *P* < 0.05 using Fisher’s protected LSD test. Incidence of different degrees of myopathies and tibial head necrosis were analyzed using a Chi-square test or the SAS GLIMMIX procedure with significant differences (*P* < 0.05) tested assuming a binomial response distribution, with a logit link function.

## Results and Discussion

Increased oxidative stress due to circulatory insufficiency and hypoxia in muscle tissue of modern broilers have been reported to be associated with the incidence of WB (Kuttappan et al., 2016; Soglia et al., 2016b; Papah et al., 2018; Sihvo et al., 2018). In fact, tissue levels of free radicals, which contribute to the oxidative stress, can be influenced by various endogenous (ischemia, inflammatory response, mitochondrial metabolism and other cytosolic sources) and exogenous (oxidized fat in diet, heat stress, ionization, radiation) factors (Jensen et al., 1997; Willcox et al., 2004; Slimen et al., 2014; Delles et al., 2015; Surai, 2015; Akbarian et al., 2016; Wang et al., 2018). Furthermore, dietary oxidized fat caused increased rancidity in meat products with concomitant increase in drip loss and reduced shelf life (Jensen et al., 1997; Delles et al., 2015). Based on this information, experiment 1 was conducted to confirm the association between oxidized fat as a source of oxidative stress on the incidence of WB and evaluate the effect of proven nutritional antioxidant intervention. As expected, oxidized fat had an impact (*P* < 0.05) on the performance of birds (Table 3) in accordance with previous reports (Dibner et al., 1996; Ao et al., 2017). A factorial analysis of live body weight for the effect of fat type showed that birds fed with oxidized fat (4.65 ± 0.04 kg) had lower (*P* < 0.05) live weight than birds fed with fresh fat (4.82 ± 0.04 kg). Previous studies have shown that presence of oxidized fat in the diet can destroy other nutrients in the diet matrix such as protein and fat-soluble vitamins (Sheehy et al., 1994; Zdunczyk et al., 2002) and increase oxidative stress related damages in tissue (Dibner et al., 1996) leading to reduced performance in broiler birds (Ao et al., 2017). However, there were no effects (*P* > 0.05) of oxidized fat on other carcass weight and yield parameters (Table 3) in the present study. The incidence of severe degree of WB (score 3) tended to be higher (*P* = 0.064; pairwise comparison) in OxF (29%) compared to FrF birds (18%). Furthermore, higher levels of plasma LDH were seen in birds fed with oxidized fat (2596.31 + 375.60 U/L) compared to fresh fat (1149.23 + 375.60 U/L) group based on factorial analysis. LDH is an enzyme present in most of the living animal cells, which is involved in metabolism of lactate (an end product of anaerobic glycolysis) and is used as an indicator of cell/tissue damage in human (Zhao et al., 2020). Previously,

**TABLE 3:** Carcass weight and yield data from Experiment 1.

	**Live weight, kg**	**Dressing %**	**Filet (kg)**	**Breast yield (%)**	**% Fat**
FrF	4.788^A^	77.08	1.164	24.32	1.45
FrF + ETX	4.855^A^	77.34	1.183	24.35	1.43
OxF	4.588^B^	77.23	1.138	24.85	1.45
OxF + ETX	4.719^AB^	76.92	1.155	24.51	1.44
SEM	0.0523	0.1470	0.0158	0.176	0.029

*P*-values from factorial analysis					
Fat Type	**0.002**	0.35	0.10	0.05	0.81
AO	0.06	0.85	0.26	0.40	0.70
Fat type*AO	0.54	0.06	0.96	0.29	0.99

*^*A,B*^Different superscript in each column differ significantly (*P* < 0.05).*

*n = 162 birds/treatment.*

*FrF, Fresh fat; FrF + ETX, Fresh fat and ethoxyquin; OxF, Oxidized fat; OxF + ETX, Oxidized fat and ethoxyquin.*

Fat type*AO means interaction between Fat type and AO.

*Bold P-values means significant (*P* < 0.05) effect.*

**FIGURE 1:**
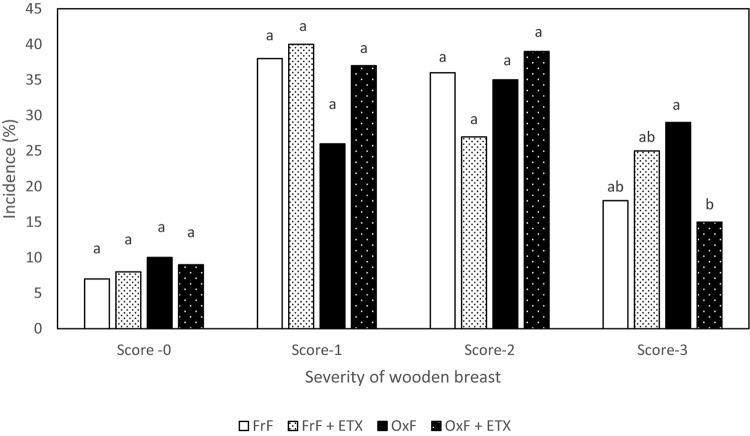
Incidence of different degrees of wooden breast in birds from Experiment 1. ^*a,b*^Different superscript under each score differ significantly (*P* < 0.05). *n* = 162 birds/treatment. FrF, Fresh fat; FrF + ETX, Fresh fat and ethoxyquin; OxF, Oxidized fat; OxF + ETX, Oxidized fat and ethoxyquin.

Kuttappan et al. (2013b) reported that increased serum levels of LDH in case of white striping myopathy, which is similar to WB, suggestive of increased muscle damage. In fact, Meloche et al., 2018a,c) reported positive association between severe WB and elevated plasma LDH. There was no difference in plasma CK levels in the present study. Meloche et al. (2018a) reported the lack of association with plasma CK and incidence of WB which could be due to non-specificity of CK to indicate muscle damage. This data confirmed that inclusion of oxidized fat in the diet is an effective exogenous way to induce muscle damage in birds. Interestingly, incidence of WB Score 3 was decreased (*P* < 0.05) in OxF + ETX (15%) group when compared to OxF (29%) group (Figure 1). A decrease (*P* < 0.05) in breast muscle TBARS was seen in ETX fed group (0.023 umol/g) when compared to non-ETX fed group (0.028 umol/g) based on factorial analysis. Soglia et al. (2016a) had reported that increased TBARS and protein carbonyls levels in muscle tissue are associated with incidence of WB in broiler birds. It is also important to point out a factorial comparison between ETX and non-ETX groups revealing a directional decrease in plasma LDH (1422.51 vs. 2323.04 U/L; *P* = 0.098) and improvement in live weight (4.787 vs. 4.688 kg/bird; *P* = 0.063) in ETX group. Nonetheless, Dibner et al. (1996) reported that inclusion of ETX in the feed had shown benefits in improving performance in broiler birds fed with oxidized fat. In short, these results confirm that dietary oxidized fat cause muscle damage as indicated by elevated LDH levels (Table 4), and ETX is effective in reducing muscle oxidative stress as shown by breast muscle TBARS levels (Table 4). Inclusion of ETX to diet with bad quality or oxidized fat significantly reduced the incidence of severe WB (Figure 1).

**TABLE 4:** Plasma and tissue parameters from Experiment 1.

	**Plasma enzymes (units per L)**		**TBARS (umol per g)**	
	**LDH**	**CK**	**Breast muscle^§^**	**Liver**
FrF	1209.52^B^	124.37	0.026^AB^	0.209^A^
FrF + ETX	1088.95^B^	125.67	0.022^B^	0.197^AB^
OxF	3436.56^A^	119.50	0.030^A^	0.182^B^
OxF + ETX	1756.06^B^	124.71	0.025^AB^	0.182^B^
SEM	530.26	2.21	0.0019	0.0088

*P*-values from factorial analysis				
Fat type	**0.0096**	0.19	0.08	**0.0224**
AO	0.10	0.15	**0.0191**	0.51
Fat type*AO	0.15	0.38	0.73	0.49

*^*A,B*^Different superscript in each column differ significantly (*P* < 0.05).*

**n* = 162 birds/treatment.*

*FrF, Fresh fat; FrF + ETX, Fresh fat and ethoxyquin; OxF, Oxidized fat; OxF + ETX, Oxidized fat and ethoxyquin.*

Fat type*AO means interaction between Fat type and AO.

*Bold P-values means significant (*P* < 0.05) effect.*

**FIGURE 2:**
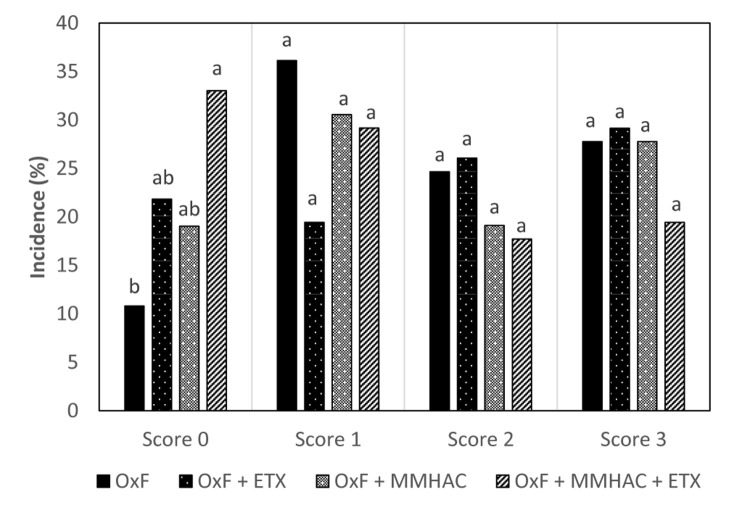
Incidence of different degrees of myopathies in birds from Experiment 2 at 42 days. ^*a,b*^Different superscript under each score differ significantly (*P* < 0.05). *n* = 36 birds/treatment. OxF, Oxidized fat; OxF + ETX, Oxidized fat and ethoxyquin; OxF + MMHAC, Oxidized fat and Zn, Cu, Mn–MMHAC; OxF + MMHAC + ETX, Oxidized fat, Zn, Cu, Mn–MMHAC, and ethoxyquin.

In Experiment 2, multiple nutritional interventions which can support the reduction of oxidative stress level in tissue were tested in the presence of oxidized fat in diet. The study compared the effect of ETX and organic source of Zn, Cu, and Mn individually or in combination, on the effect of tissue oxidative stress level and incidence of WB. Results from the study showed that the individual or combined addition of ETX with MMHAC resulted in improved (*P* < 0.05) cFCR when compared to OxF (Table 5). Both, OxF + ETX, and OxF + MMHAC + ETX were able to improve (*P* < 0.05) the cPERFIDX when compared to OxF (Table 5). OxF + MMHAC + ETX resulted in an additive effect with an improvement (*P* < 0.05) in incidence of normal filets when compared to OxF (Figure 2). Interestingly, OxF + MMHAC + ETX showed the lowest (*P* < 0.05) level of breast muscle TBARS among the treatments in the study. Superoxide radicals, contributing to increased oxidative stress and higher TBARS levels in tissues, are primarily produced in mitochondria by the electron transport chain and in the cytoplasm by enzymes such as xanthine oxidase, cytochrome P450-monooxygenases, NADPH oxidase (Wang et al., 2018). Once produced in mitochondria, superoxides can move to cytoplasm where they can move back and forth to the extracellular space (Wang et al., 2018). These superoxides can then be reactive with other molecules to form reactive oxygen species which can cause damage to DNA, RNA, proteins, and lipids in the cell (Surai, 2015). The antioxidant system present in the body plays an important role to convert the superoxide to harmless substances. Primarily, superoxides are converted to hydrogen peroxide by a group of enzymes called superoxide dismutases (SOD). Surai (2015) reported there are mainly three types of SODs found in mammals and chickens. Mn-SOD (mitochondria), Zn, Cu-SOD (cytoplasm), and Cu-SOD (extracellular space) which need the respective trace minerals as cofactors for proper functioning (Surai, 2015; Wang et al., 2018). According to Surai (2015), the activity of these SOD in tissues can be enhanced by adequate bioavailable levels of Zn, Cu, and Mn in diet. In fact, MMHAC trace minerals have been well studied for their improved bioavailability compared to ITMs (Yan and Waldroup, 2006; Wang et al., 2007). Furthermore, the enhanced tissue activity of SOD can increase conversion of oxygen free radicals to hydrogen peroxide which in turn is acted upon by catalase and glutathione peroxidase to water (Surai, 2015; Wang et al., 2018), thereby reducing the damage from oxidative stress. A combination of ETX and MMHAC–Zn, Cu, Mn in experiment 2 could be reducing the effect of both exogenous (dietary oxidized fat) and endogenous (superoxides in mitochondria, cytoplasm, and extracellular space) factors to cause oxidative stress and thereby provided a greater magnitude of reduction in tissue oxidative stress and severity of WB along with improving performance in broiler birds. In addition, Richards et al. (2010) reported that Zn, Cu, and Mn play an important role in the development of connective tissue and tissue repair. Plausibly, this benefit could also be contributing to the advantage of MMHAC reducing severity of muscle damage leading to WB. However, the treatments only showed numerical reduction in serum LDH levels in this study suggesting of a tendency to reduce muscle damage (Table 5).

**TABLE 5:** Performance data and tissue parameters from birds from Experiment 2.

	Performance* (*n* = 18 pens/treatment; 15 birds/pen)				**Tissue parameters (36 birds/treatment)**	
	**BW (kg)**	**cFCR (kg:kg)**	**cFI (kg)**	**cPerfidx**	**Serum LDH (milliunits/mL)**	**Breast muscle TBARS (mmol/g)**
OxF	3.807	1.564^A^	5.891	504.580^B^	1690.44	0.038^AB^
OxF + ETX	3.906	1.537^B^	5.941	529.600^A^	1397.47	0.033^BC^
OxF + MMHAC	3.868	1.540^B^	5.897	519.985^AB^	1305.15	0.043^A^
OxF + MMHAC + ETX	3.861	1.539^B^	5.879	521.465^A^	1560.95	0.027^C^
SEM	0.0287	0.0044	0.0445	5.8773	161.95	0.002
*P*-value	0.11	**< 0.0001**	0.77	**0.0257**	0.34	**0.0001**

^*A,B*^Different superscript in each column differ significantly (*P* < 0.05).

*Pens were used as experimental units for performance parameters.

*OxF, Oxidized fat; OxF + ETX, Oxidized fat and ethoxyquin; OxF + MMHAC, Oxidized fat and Zn, Cu, Mn–MMHAC; OxF + MMHAC + ETX, Oxidized fat, Zn, Cu, Mn–MMHAC, and ethoxyquin. Bold numbers means *P* < 0.05.*

**FIGURE 3:**
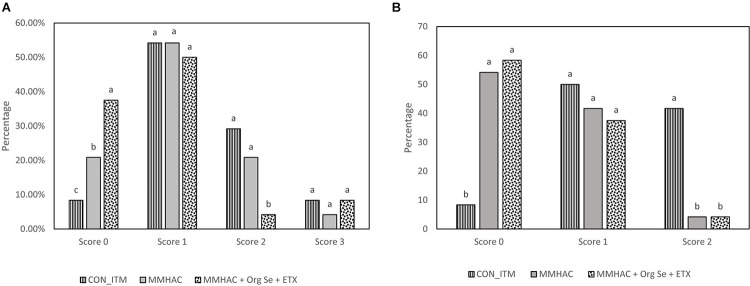
Incidence of different degrees of WB **(A)** and tibial head lesions **(B)** in birds from Experiment 3. ^a–c^Different superscript under each score differ significantly (*P* < 0.05). *n* = 24 birds/treatment. CON-ITM with sulfate of zinc (110 ppm), copper (6 ppm), and manganese (120 ppm) with inorganic selenium (0.3 ppm). MMHAC with MMHAC sources of zinc (50 ppm), copper (10 ppm), manganese (60 ppm) with inorganic selenium (0.3 ppm). MMHAC + Org Se + ETX with MMHAC sources of zinc (50 ppm), copper (10 ppm), manganese (60 ppm) with organic selenium (ZORIEN^®^ SeY, Novus International, Inc., added at 0.3 ppm) and AO at 125 ppm of final concentration.

**FIGURE 4:**
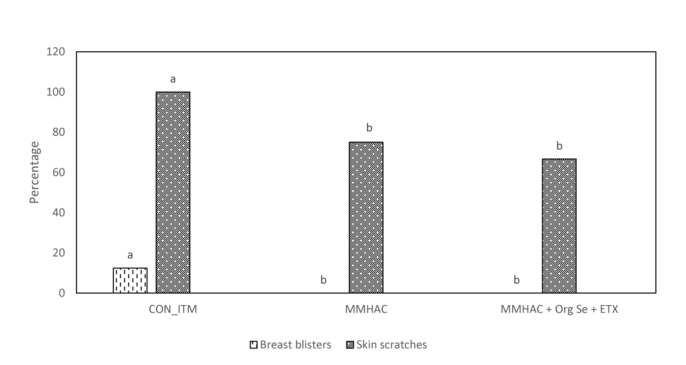
Incidence of breast blisters and skin scratches in birds from Experiment 3. ^*a,b*^Different superscript under each score differ significantly (*P* < 0.05). *n* = 24 birds/treatment. CON-ITM with sulfate of zinc (110 ppm), copper (6 ppm), and manganese (120 ppm) with inorganic selenium (0.3 ppm). MMHAC with MMHAC sources of zinc (50 ppm), copper (10 ppm), manganese (60 ppm) with inorganic selenium (0.3 ppm). MMHAC + Org Se + ETX with MMHAC sources of zinc (50 ppm), copper (10 ppm), manganese (60 ppm) with organic selenium (ZORIEN^®^ SeY, Novus International, Inc., added at 0.3 ppm) and AO at 125 ppm of final concentration.

In Experiment 3, various nutritional intervention strategies to reduce oxidative stress in tissue were combined and tested in a commercial relevant situation where birds were fed a corn-soy based diet with no oxidized fat but exposed to mild heat stress during the grow out period to mimic environmental conditions that could lead to oxidative stress (Slimen et al., 2014; Akbarian et al., 2016; Lu et al., 2017). The harmful effects of heat stress in birds could be attributed through disruption of mitochondrial function, increased oxidative stress and lipid peroxidation, decreased vitamin levels and altered enzyme activity leading to reduced production performance (Horváth and Babinszky, 2018). In this study, there were no differences (*P* > 0.05) between the treatment groups with respect to live weight, or carcass weight and yield (Table 6). However, MMHAC treatment resulted an increase (*P* < 0.05) in the incidence of normal filets (Score 0) with respect to CON-ITM (Figure 3A). Previously, Sirri et al. (2016) reported that use of MMHAC resulted in numerical improvement in incidence of WB score 0 (MMHAC 88% vs. ITM 84%) and the lack of significant difference could be due to overall lower incidence of WB in that study. In fact, the results from Experiment 1 showed the effect of these intervention strategies will be more prominent when there is higher challenge. In addition, it was observed in Experiment 2 that combining MMHAC + ETX provided a higher magnitude of benefit in reducing oxidative stress in muscle and increasing percentage of normal (no WB) fillets. One step further in Experiment 3, adding Org Se and ETX to MMHAC resulted in a higher magnitude of increase (*P* < 0.05) in normal filets and a reduction (*P* < 0.05) in Score 2 WB when compared to both CON-ITM and MMHAC groups (Figure 3A). Selenium is a cofactor for glutathione peroxidase which converts hydrogen peroxide to water (Slimen et al., 2014; Surai, 2015). Kumbhar et al. (2018) reported that dietary combination of Se and Vitamin E (rather than used individually) was effective in increasing the catalase, SOD, as well as glutathione peroxidase activities thereby reducing malondialdehyde level or oxidative stress in breast muscle tissue from birds under heat stress. Horváth and Babinszky (2018) reported that high levels of vitamin A, E, C, and Zn as well as Se can support enzyme activity, reduce oxidative stress, and improve production performance in birds under heat stress. Interestingly, the antioxidant benefits from vitamins and microminerals were greater when combined in diets for birds under heat stress (Horváth and Babinszky, 2018). When different dietary interventions to reduce oxidative stress are combined, the benefits could be expressed at different points and sources of oxidative stress such as, at dietary fat sources, during cellular level conversion of superoxides to hydrogen peroxide and then to water in mitochondria, cytoplasm as well as extracellular space. The multi-step cellular oxidative stress process could be the reason why the magnitude of response is higher when combined than when supplemented individually. In addition, inclusion of MMHAC–Zn, Cu, Mn with or without Org Se and ETX resulted in an increase (*P* < 0.05) in normal tibial bones and a decrease (*P* < 0.05) in tibia with severe bacterial necrosis lesions (Figure 3B). According to Wideman and Prisby (2013), bacterial necrosis of bone can occur through the translocation of bacteria from gut or respiratory tract to the bone along with microdamages happening in the bone tissue, resulting in colonization of bacteria in bone marrow. Results from the present study also showed that the inclusion of MMHAC–Zn, Cu, Mn in the diet resulted in numerical improvement although MMHAC + Org Se + ETX showed significant (*P* < 0.05) improvement in tibial strength (Table 6), thus preventing the occurrence of microdamage leading to bacterial necrosis. Dibner et al. (2007) had reported that feeding a combination of MMHAC Zn, Cu, and Mn in broiler birds can enhance the cortical thickness and improve the breaking strength in tibia which agrees with the present study. Furthermore, Sirri et al. (2016) showed similar results where the use of Zn, Cu, Mn–MMHAC showed lower femoral and tibial lesions when compared to ITMs. Interestingly, inclusion of MMHAC and antioxidants in the diet also resulted in decreased breast blisters and skin scratches (Figure 4). In addition to the benefits of Zn, Cu, and Mn in the integrity of connective tissue and tissue repair (Richards et al., 2010; Chen et al., 2017), zinc plays an important role in the wound healing process and feathering in birds (El-Hack et al., 2017). Thus, the benefits of MMHAC on reducing carcass skin lesions could be due to greater skin and feather integrity and/or faster recovery and wound healing (Chen et al., 2017) from scratches and blisters (because of the enhanced tissue repair).

**TABLE 6:** Live weight, carcass weight as well as yield and tibial breaking strength of birds from Experiment 3.

	**Live weight, kg**	**Dressing %**	**Filet (g)**	**Breast yield (%)**	**% Fat**	**Tibial strength (kg/cm^2^)**
CON-ITM	2.563	76.90	550	27.90	2.27	0.34^B^
MMHAC	2.611	77.15	577	28.66	2.16	0.37^AB^
MMHAC + Org Se + ETX	2.586	76.79	563	28.36	2.15	0.41^A^
SEM	0.02	0.27	8.2	0.26	0.08	0.02
*P*-value	0.54	0.06	0.96	0.29	0.99	**<0.01**

*^A,B^Different superscript in each column differ significantly (*P* < 0.05). *n* = 24 birds/treatment.*

• CON-ITM with sulfate of zinc (110 ppm), copper (6 ppm), and manganese (120 ppm) with inorganic selenium (0.3 ppm).

• MMHAC with MMHAC sources of zinc (50 ppm), copper (10 ppm), manganese (60 ppm) with inorganic selenium (0.3 ppm).

• MMHAC + Org Se + ETX with MMHAC sources of zinc (50 ppm), copper (10 ppm), manganese (60 ppm) with organic selenium (ZORIEN^®^ SeY, Novus International, Inc., added at 0.3 ppm) and AO at 125 ppm of final concentration. Bold numbers means *P* < 0.05.

## Conclusions

The factors that increase the incidence of WB are not clearly known, however circulatory insufficiency and increased oxidative stress in the breast muscles is reported to be associated with WB. The results from the first experiment showed that presence of oxidized fat in the diet can reduce performance in birds and increase muscle damage. Interestingly, inclusion of ETX to diet with oxidized fat reduced the incidence of severe WB in broilers. Based on the muscle TBARS levels, ETX was effective in reducing oxidative stress in muscle tissue. In experiment 2, combination of MMHAC (Zn, Cu, and Mn) chelated trace minerals with ETX reduced oxidative stress in muscle tissue and increased the percentage of normal (without WB) filets in birds fed diet with oxidized fat. In the third experiment under mild heat stress, feeding MMHAC alone reduced incidence of tibial head lesions, skin scratches, breast blisters, and increased the incidence of normal fillets. When MMHAC was combined with ETX and Org Se, further increase in the incidence of normal filets were observed. The combination of ETX, chelated trace minerals in the form of MMHAC, and Org Se resulted in the reduction of oxidative stress in the tissue plausibly through activation of endogenous antioxidant enzymes and reducing dietary sources of oxidative stress. Nonetheless, MMHAC could provide additional benefits in improving the connective tissue integrity and enhanced tissue repair in muscle, skin, and bone. Further studies are needed to understand the mode of action of these dietary interventions in reducing carcass quality defects such as myopathies.

## Data Availability Statement

The original contributions presented in the study are included in the article/supplementary material, further inquiries can be directed to the corresponding author/s.

## Ethics Statement

The animal study was reviewed and approved by Animal Use Committee, Novus International, Inc., Green Acres Experimental Farm, Montgomery, Missouri Internal Ethics Committee, at Bangkok Animal Research Center Co., Ltd., Thailand.

## Author Contributions

VK, MM, and MB carried out the project and data analysis, while JC supported with the tissue analysis. VK put together the information, interpreted the results, and prepared the manuscript under the supervision of MV-A. All authors contributed to experimental design, discussed the results, and reviewed the manuscript.

## Conflict of Interest

All the authors are employed by Novus International, Inc. and declare that the research was conducted in the absence of any commercial or financial relationships that could be construed as a potential conflict of interest.
